# Differential Effects of Four Canonical Notch-Activating Ligands on c-Kit+ Cardiac Progenitor Cells

**DOI:** 10.3390/ijms252011182

**Published:** 2024-10-17

**Authors:** Matthew Robeson, Steven L. Goudy, Michael E. Davis

**Affiliations:** 1 Wallace H. Coulter Department of Biomedical Engineering, Georgia Institute of Technology and Emory University, Atlanta, GA 30332, USA; matthew.robeson@emory.edu; 2 Department of Pediatric Otolaryngology, Emory University, Atlanta, GA 30322, USA; steven.goudy@emory.edu; 3 Children’s Heart Research and Outcomes (HeRO) Center, Emory University and Children’s Healthcare of Atlanta, Atlanta, GA 30322, USA

**Keywords:** stem cells, Notch signaling, cardiac regeneration

## Abstract

Notch signaling, an important signaling pathway in cardiac development, has been shown to mediate the reparative functions of c-kit+ progenitor cells (CPCs). However, it is unclear how each of the four canonical Notch-activating ligands affects intracellular processes in c-kit+ cells when used as an external stimulus. Neonatal c-kit+ CPCs were stimulated using four different chimeric Notch-activating ligands tethered to Dynabeads, and the resulting changes were assessed using TaqMan gene expression arrays, with subsequent analysis by principal component analysis (PCA). Additionally, functional outcomes were measured using an endothelial cell tube formation assay and MSC migration assay to assess the paracrine capacity to stimulate new vessel formation and recruit other reparative cell types to the site of injury. Gene expression data showed that stimulation with Jagged-1 is associated with the greatest pro-angiogenic gene response, including the expression of VEGF and basement membrane proteins, while the other canonical ligands, Jagged-2, Dll-1, and Dll-4, are more associated with regulatory and epigenetic changes. The functional assay showed differential responses to the four ligands in terms of angiogenesis, while none of the ligands produced a robust change in migration. These data demonstrate how the four Notch-activating ligands differentially regulate CPC gene expression and function.

## 1. Introduction

Loss of contractile function due to cardiomyocyte death and adverse remodeling following a myocardial infarction (MI) is a major cause of heart failure. Because of the limited availability of transplants, and the frequent presence of comorbidities that make MI survivors poor candidates for transplantation, research into cardiac regenerative medicine in recent years has focused heavily on cell-based therapies, with the goal of augmenting the body’s natural capacity for repair and revascularization of the heart following an injury, hopefully negating the need for transplantation in patients whose treatment options are otherwise limited.

One population of cells that has been extensively studied for this purpose both in vitro and in clinical trials is c-kit+ cardiac progenitor cells (CPCs), a small population of stem cells endogenous to the cardiac niche that have been shown to home to the site of injury following an MI [1]. Though CPCs do not have the capacity to form new cardiomyocytes, research from our lab and others has shown that these cells contribute to a pro-reparative response by way of pro-angiogenic paracrine release and differentiation into a vascular endothelial lineage, contributing to revascularization around the infarct [2,3,4,5,6]. The first large-scale clinical trial of CPCs in patients with heart failure, the CONCERT-HF trial, demonstrated that CPCs injected into the myocardium elicited better clinical outcomes than placebo, including an 80% reduction in major adverse cardiac events compared to the placebo group [7]. Though strong evidence supports the pro-reparative capacity of CPC therapy, the important factors driving this response are not entirely understood. One particular shortcoming of cell-based therapies is the difficulty of recreating important chemical and biological signaling cues present in the cardiac niche [1,5,6]. Specifically in CPCs, the Notch signaling pathway, important in cardiac growth and development, has been shown to have a profound effect on the pro-reparative capacity of CPCs [8,9]. However, Notch signaling is a complex and intricately regulated pathway, involving multiple receptor and ligand pairings that can elicit different—and sometimes even opposite—responses. This warrants further exploration into the effects of stimulation with different Notch-activating ligands on CPCs.

Notch signaling is a juxtacrine signaling pathway, meaning that signaling is initiated when a ligand-presenting cell comes into physical contact with a receptor-presenting cell. The Notch receptor, of which there are four canonical isotypes, is a single-pass transmembrane receptor with an exocellular domain, a transmembrane domain, and an intracellular domain [10]. Upon ligand binding, a pulling force pulls apart the Notch receptor, exposing a protease cleavage site within the transmembrane domain that normally exists in an autoinhibited state. γ-secretase, a transmembrane protease complex, then cleaves the Notch receptor at this exposed site, releasing the Notch intracellular domain (NICD), which translocates to the nucleus where it acts as a potent transcriptional regulator on numerous downstream targets [11]. The unusual requirement of a pulling force to activate Notch, sometimes described as a form of ‘mechanical allostery’, presents a technical challenge when trying to activate Notch signaling in a cell population in vitro [11]. Soluble Notch-activating ligands are not sufficient to activate Notch, and in fact inhibit the activation of Notch since they are able to bind to the Notch receptor, but the lack of a pulling force does not expose the autoinhibited cleavage site. Some studies have suggested that endocytosis of the ligand by a ligand-presenting cell is indispensable for Notch activation [12,13]. However, our studies and others have shown that immobilization of Notch-activating ligands on a surface or material is sufficient to activate Notch. This has been accomplished in a few ways, including coating surfaces with Notch-activating ligands or engineering hydrogels to present Notch-activating ligands. For the purposes of this study, we chose to adapt a method published by Kamalakar et. al., in which Notch ligands conjugated to the Fc-region of IgG are bound by magnetic Dynabeads [14]. Unlike materials for 3D culture, such as hydrogels, this technique elicits a strong response while using minimal amounts of ligands.

Notch signaling is critical in a wide variety of biological processes, including cardiac development, where the inheritance of a defective copy of the Notch receptor is associated with several congenital heart defects [8,15]. Most importantly, for our purposes, however, Notch signaling intimately regulates the process of angiogenesis in vascular endothelial cells. Because CPCs are an endothelial lineage cell type, it stands to reason that Notch signaling may play a similarly important role in regulating CPC fate. The four canonical Notch ligands—Jagged1, Jagged2, and the Delta-like ligand family, Dll1 and 4—play different roles in the process of angiogenesis. The process of vessel sprouting begins when a VEGF gradient within the extracellular matrix stimulates the selection of a tip cell, the cell that will serve as the advancing front of the newly formed vessel [16]. Tip cell selection drives higher expression of Dll4, which acts on neighboring cells to suppress the tip cell phenotype. These cells become the stalk cells, which make up the lumen of the newly formed vessel. Stimulation with Jagged1, however, acts antagonistically to this Dll4 signal, suppressing the stalk cell phenotype and stimulating the formation of new tip cells further down the stalk, leading to increased vessel branching.

Though two previous studies in our lab have demonstrated that stimulating Notch signaling in CPCs in vitro improves their reparative capacity, a few important questions still remain unanswered. The first of these studies examined the stimulation of CPCs in a self-assembling peptide-based hydrogel with tethered ligands; however, this study only looked at stimulation with Jagged-1. The second study took a different approach, using spherical aggregation to stimulate Notch signaling by natural cell-to-cell signaling, but this study also did not consider the relative effects of stimulation with different ligands. The goal of the present study, therefore, is to determine if stimulation of CPCs by the different canonical Notch ligands drives a substantially different response, whether that response mirrors phenotypic changes observed in endothelial cells, and whether stimulation with Jagged-1 as we have performed previously is the best option for improving functional outcomes.

## 2. Results

### 2.1. Treatment with Notch Ligand Functionalized Beads Induces Fluorescence in YFP-Notch CHO Cells

**Figure 1:**
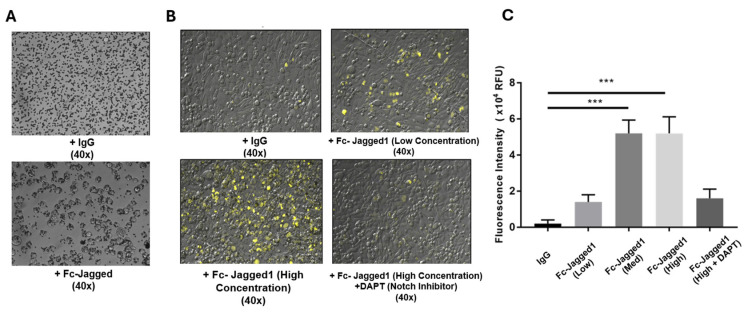
Fc-Jagged functionalized beads bind and activate Notch in vitro. (**A**) Fc-Jagged1 functionalized beads aggregate on the surface of c-kit+ hCPCs compared to nonspecific human IgG functionalized beads. (**B**,**C**) Fluorescent YFP is expressed when cells are stimulated with Jagged-1 beads. The response is attenuated when the small molecule Notch inhibitor DAPT (10 µmol/L) is added to cell media. N = 6 per group. (***) *p* < 0.005, one-way ANOVA.

To demonstrate the effectiveness of Jag-1 functionalized beads in activating Notch signaling in vitro, we utilized a reporter cell line, consisting of CHO cells engineered to express YFP upon activation of Notch, as previously reported by Elowitz et al. [17]. In this reporter cell line, YFP expression is driven by the cleavage of the Notch receptor, which includes an attached, truncated variant of the transcriptional activator Gal4, which in turn drives YFP expression under the control of a UAS promoter. This approach allows for easy visual feedback to verify Notch activation without needing to probe gene or protein targets through PCR or Western blotting, which in turn allows for easy troubleshooting of the study design. These reporter cells were incubated for 48 h with Jag1 functionalized Dynabeads before fluorescence was assessed using confocal microscopy. ImageJ was then used to quantify mean fluorescence across the entire frame. Three serial dilutions of functionalized beads were tested, termed low, medium, and high concentrations, as shown in Figure 1. All concentrations of beads demonstrated an increase in fluorescence upon treatment, with the maximum signal being attained at the medium concentration, corresponding to approximately 0.5 µg of Fc-Jagged per 250,000 cells. Based on this result, this concentration was chosen for all future experiments. Additionally, it was shown that DAPT (10 µM), a small molecule inhibitor of Notch that works by deactivating the γ-secretase complex, was able to attenuate the fluorescence response such that there was no significant difference in the DAPT-treated group and the nonspecific IgG control group. Thus, the response was significant to stimulation with Jagged and not a nonspecific effect.

### 2.2. c-Kit+ hCPCs Demonstrate Different Gene Expression Profiles upon Treatment with Different Canonical Notch Ligands

**Figure 2:**
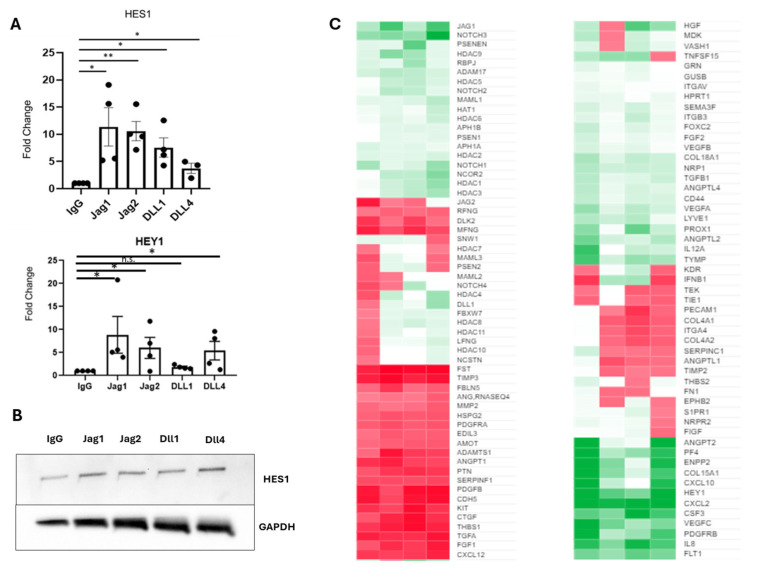
The four canonical Notch ligands drive substantially different gene expression profiles in c-kit+ hCPCs. (**A**) HES1 expression is upregulated in all four treatment groups, though Jag1 and Jag2 drive the strongest response. HEY1 expression is upregulated by Jag1, Jag2, and Dll4, but not Dll1. N = 4 per group, (**) *p* < 0.005, (*) *p* < 0.05, (n.s.) not significant, one-way ANOVA. (**B**) HES1 protein level increases upon activation of Notch by Jag1/Jag2/Dll1/Dll4. (**C**) Heat map demonstrating gene expression in response to all 4 ligands. Gene expression was normalized to IgG control beads and presented as fold change over control.

As a broad metric of Notch activation in c-kit+ hCPCs, the two immediate downstream transcriptional activators, HES1 and HEY1, were selected. As shown in Figure 2A, treatment with all Notch-activating ligands stimulated an increase in HES1 expression, ranging from 4-fold to 10-fold, although Jag1 and Jag2 elicited the strongest response. Curiously, although Jag1, Jag2, and Dll4 were able to elicit the expression of HEY1, Dll1 did not elevate the expression of HEY1 compared to the nonspecific IgG control. This increase in HES1 was verified by Western blot, as shown in Figure 2B.

To further probe the differences in gene expression profiles among the four treatment groups, two different TaqMan gene arrays were utilized, the combined results of which are shown in Figure 2C.

Several interesting observations can be gleaned from the results of the gene array. In particular, Jag1 drives a different response than the other three canonical ligands in several key clusters of genes. For instance, the histone deacetylase and Mastermind (MAML) class of genes, associated with epigenetic remodeling, is broadly downregulated by Jag1 but modestly upregulated in the other three treatment groups. The cluster of genes, including PECAM1 and several collagen subtypes most associated with extracellular matrix remodeling, is modestly upregulated by stimulation with Jag1, while it is moderately downregulated by the other three canonical ligands. Finally, pro-angiogenic markers, including ANGPT2, FLT1, IL8, and VEGFC, are much more strongly upregulated by Jag1 as compared to the other three canonical ligands.

**Figure 3:**
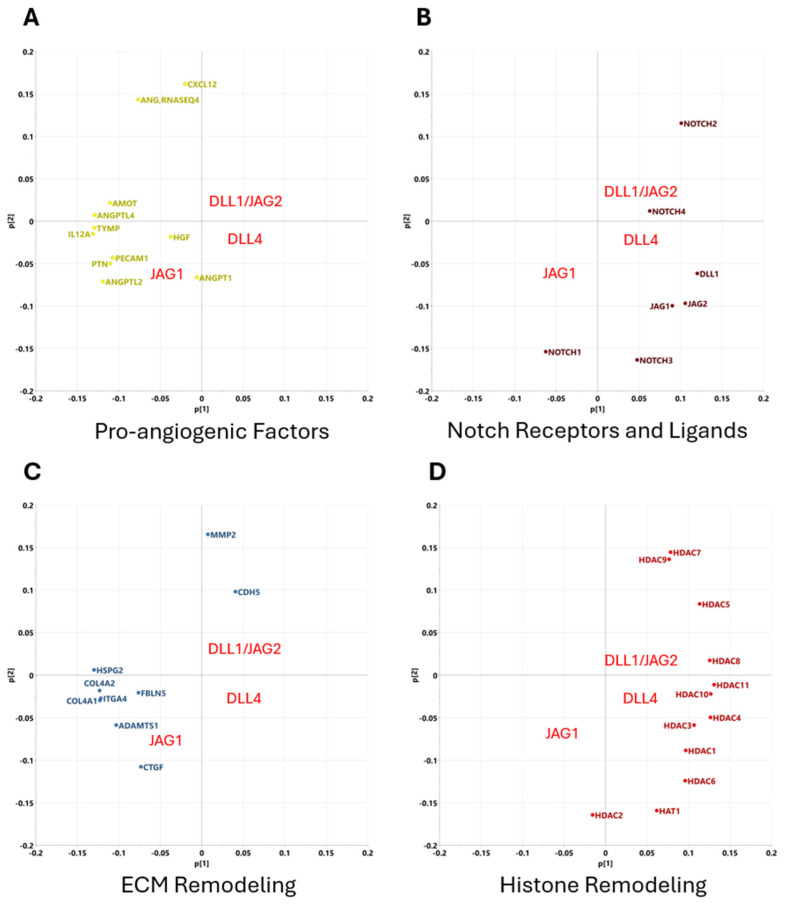
PCA reveals clusters of genes that co-vary closely with particular Notch-activating ligands. (**A**) Genes associated with pro-angiogenic factors co-vary with Jag1 stimulation. (**B**) Genes associated with ECM remodeling co-vary with Jag1 stimulation. (**C**) Genes associated with Notch receptors and ligands co-vary with Jag2/Dll1/Dll4 stimulation. (**D**) Genes associated with Hitone remodeling co-vary with Jag2/Dll1/Dll4 stimulation.

In order to better visualize clusters of genes that co-vary with particular Notch ligands, principal component analysis was utilized in order to reduce the dimensionality of the dataset and visualize relationships between ligands and associated genes. The genes were categorized based on their primary function, including pro-angiogenic factors, epigenetic regulation, and ECM remodeling. Principal component analysis is an unsupervised learning technique by which a large dimensionality dataset is reduced into its two primary components, which are then projected on a 2D plane. This type of analysis allows us to better visualize the clusters of genes that co-vary according to each ligand treatment. Figure 3 shows the results of the principal component analysis.

When genes are categorized based on their function, we find that these genes are separated on opposite ends of the x-axis, corresponding to stimulation with either Jag1 or Jag2/Dll1/Dll4, supporting our hypothesis that Jag1 drives a substantially different response than the other canonical ligands. Pro-angiogenic factors as well as genes associated with remodeling of the ECM cluster to the left-hand side of the x-axis, corresponding to stimulation with Jag1. Likewise, genes associated with epigenetic remodeling, such as the HDAC family, as well as the Notch ligands and receptors themselves, tend to cluster to the right-hand side of the x-axis, corresponding to stimulation with Jag2/Dll1/Dll4. This further supports our hypothesis that, like in endothelial cells, Jag1 drives a more active, angiogenic phenotype among CPCs, while the other three canonical ligands serve more of a regulatory role.

### 2.3. Secreted Factors from Notch-Stimulated c-kit+ hCPCs Upregulate Angiogenesis in an Endothelial Cell Tube Formation Assay, but Differences between Treatment Groups Are Non-Significant

**Figure 4:**
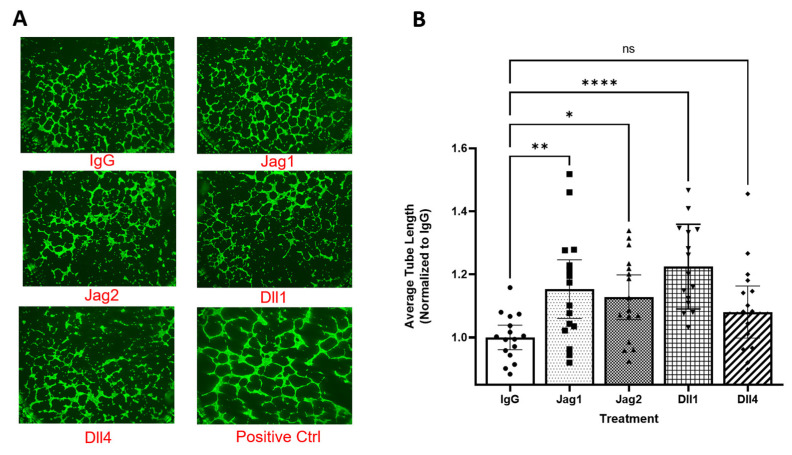
Endothelial cell tube formation is enhanced across all treatment groups compared to IgG, but differences between the four canonical ligands are not significant. (**A**) Representative fluorescent images of endothelial cell tubes after 6 h. (**B**) Quantified average tube length. N = 16 for each treatment group. (*) *p* < 0.05, (**) *p* < 0.005, (****) *p* < 0.00005, (ns) Not significant, one-way ANOVA.

In order to test the hypothesis that Jag1 stimulation drives a stronger release of pro-angiogenic factors to the surrounding environment, an endothelial cell tube formation assay was performed in which conditioned media from stimulated hCPCs was used to treat cardiac endothelial cells on a Matrigel substrate, as previously described. Contrary to our hypothesis, although all groups demonstrated an increase in average tube length between 15 and 20% when compared to treatment with control media treated with nonspecific IgG alone, Jag1 did not increase average tube length when compared with the other three canonical ligands. Results of this assay are shown in Figure 4. 

### 2.4. Secreted Factors from Notch-Stimulated c-kit+ hCPCs and the Migration of MSCs, but Differences between Treatment Groups Are Non-Significant

**Figure 5:**
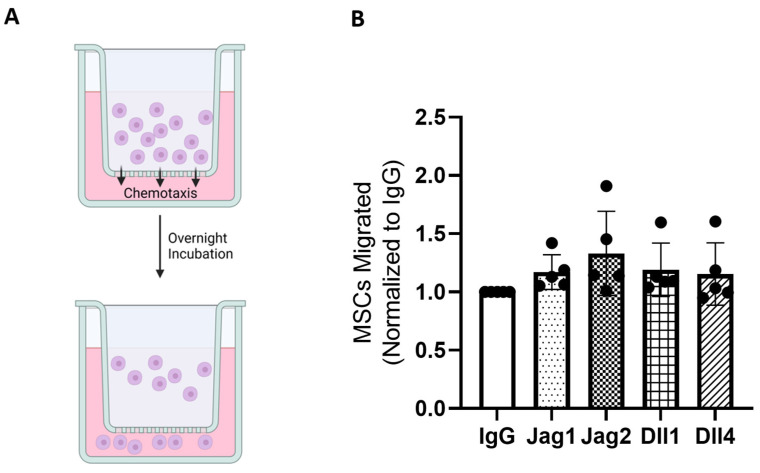
MSC migration in response to conditioned media is upregulated across all treatment groups, though differences between groups are non-significant. (**A**) MSCs are seeded with serum-free media in the upper well of a transwell insert, with conditioned media positioned below. Migration is quantified after overnight incubation. (**B**) MSC migration quantified by fluorescence measurement.

As with tube formation, we hypothesized that conditioned media from cells stimulated with Jag1 would stimulate the migration of mesenchymal stem cells (hMSCs) via a transwell assay. We based this hypothesis on two factors. First, the literature has shown a high degree of interplay between MSC and CPC populations, with MSCs shown in multiple studies to both recruit and stimulate CPCs [18,19]. We hypothesize that this axis likely works both ways, with CPCs able to also recruit and stimulate MSCs, which are highly important to the endogenous repair mechanism for the heart. Supporting this hypothesis, we note that two chemokines known to influence MSC migration, CXCL2 and CXCL10, are highly upregulated, most significantly in response to treatment with Jag1. We, therefore, performed a transwell migration assay using conditioned media to assess whether secreted factors in CPC conditioned media can influence the migration of MSCs, as shown in Figure 5.

While we noted a broad upregulation of MSC migration in response to treatment with all ligands, again, the difference between groups was not significant. This demonstrates that Notch activation in CPCs likely induces a pro-migratory release of paracrine factors, but specific ligand choice may not be an important factor.

## 3. Discussion

Several previous studies in the literature have set out to understand Notch ligand discrimination in vivo and how stimulation with the four different canonical Notch ligands can drive different programs of gene expression. A study by Elowitz et al. demonstrated, for instance, that stimulation of an engineered reporter cell line with Dll1 and Dll4 produces differing levels of Hey1 and Hes1, with Dll1 primarily mediated through Hes1 activity and Dll4 expressing both Hey1 and Hes1 [20]. Our results demonstrate the same pattern of expression, with Dll1 stimulation strictly upregulating Hes1. Elowitz et al. demonstrated that this effect is due to temporal changes in Notch receptor activation, with Dll1 inducing a pulsatile signal while Dll4 drives a steady level of Notch activation over a longer period of time. This temporal regulation, and the ratio of Hey1/Hes1 expression, drive different programs of downstream gene expression [20]. Based on this, we hypothesized that hCPCs would also demonstrate ligand discrimination, with a distinctly different response driven by each ligand. Our goal was to determine which ligand would produce the best phenotype for cardiac repair following injury.

Though hCPCs have been shown to be an endothelial-like lineage, this has not necessarily provided any clues as to how hCPCs will respond to different Notch signals, as even atrial and ventricular endothelial cells have been shown to demonstrate different transcriptional programs in response to stimulation with the same ligand [21]. Broadly speaking, within endothelial cells, Notch signaling has been shown as the primary regulator of tip and stalk cell specification, the process by which new vessel sprouting occurs [21]. Specifically, Jag1 and Dll4 have been shown to have antagonistic roles in this process. Jagged1 induces a tip cell phenotype, increasing vessel sprouting and promoting the formation of new vessels in response to a gradient of VEGF [22]. Conversely, Dll4 acts antagonistically, negatively regulating the formation of new tip cells and suppressing the branching of new vessels. The competing level activity of Jag1 and Dll4 is also closely mediated by the family of glycosyltransferases known as fringe. High fringe activity glycosylates the Notch receptor, increasing its affinity for Dll4 and suppressing activation by Jag1, thereby inhibiting the branching of new tip cells. Our observations of the transcriptome of hCPCs upon stimulation with the different canonical Notch ligands largely align with the literature on endothelial cells. The expression of all three fringe subtypes (LFNG, RFNG, and MFNG) is strongly and specifically downregulated in response to Jag1 stimulation, suggesting that Jag1 not only induces a tip cell phenotype within hCPCs but promotes reduced glycosylation of the Notch receptors on neighboring cells, promoting further activation by Jag1 and inhibiting Dll4 signaling, which would steer the cells to a more quiescent stalk cell phenotype.

hCPCs also likely act on endothelial cells within the native tissue by way of secreted factors. Jag1 induces the greatest expression of all three vascular endothelial growth factor (VEGF) isotypes that were quantified, particularly VEGFC, which was five-fold upregulated in response to Jag1 stimulation as opposed to two- to three-fold upregulated upon stimulation with the other ligands. While VEGFA is primarily responsible for the intramyocardial formation of vessels, VEGFC plays a more specific role in promoting vessel growth in the epicardium [23]. Jag1, therefore, shows a broader potential to affect angiogenesis in multiple regions within the failing heart. Jag1 also likely contributes to angiogenesis through remodeling of the extracellular matrix. Stimulation with Jag1 increased the expression of several collagen subtypes, including COL4A1, COL4A2, COL15A1, and COL18A1. Jag1 stimulation elicited the greatest expression of all four collagen subtypes tested, particularly COL4A1 and COL4A2, where stimulation with the other three canonical ligands showed strong downregulation of these collagen subtypes. COL4A1 and COL4A2 are important angiocrine genes, constituting necessary components of the basement membrane. Secretion of new basement membrane proteins is known to precede the formation of new vasculature [24].

With these observations in mind, we performed an endothelial cell tube formation assay to determine if the pro-angiogenic gene expression associated with Jag1 stimulation translated to enhanced vessel formation in vitro. While all four ligands demonstrated enhanced vessel formation when compared to a control of fresh, serum-free media, there were no significant differences noted between the four ligands, despite differing levels of response. There are a couple of hypothesized reasons as to why this result may not mirror what happens in vivo. Since tube formation assays are performed on an already well-developed basement membrane substitute (Matrigel), the secretion of collagens, including Col4A1 and Col4A2, likely does not contribute to angiogenesis in this assay, unlike in vivo conditions where the secretion of basement membrane proteins precedes new vessel formation. Additionally, the cardiac endothelial cells used in this assay may respond primarily to VEGFA, the primary mediator of vessel formation within the bulk of the myocardium. VEGFC, which is more strongly expressed in the Jag1-stimulated group, may have beneficial effects in vivo that are not recapitulated in vitro.

Another potentially interesting finding relates to the expression of two genes involved in fibrosis following an injury to the myocardium, TIMP2 and ADAMTS1. The dysregulated activity of endogenous metalloproteinases within the heart following an injury is linked with adverse remodeling and fibrosis of the heart. The previous literature has shown that the expression of tissue inhibitors of metalloproteinases (TIMP2) can exert an anti-fibrotic effect [25]. According to the results of our gene array, TIMP2 is modestly upregulated in response to stimulation with Jag1, while stimulation with the other canonical ligands results in a downregulation of TIMP2, suggesting that Jag1 treatment may be the most beneficial in promoting an anti-fibrotic phenotype in hCPCs. Additionally, of interest is the metalloproteinase ADAMTS1, which is found to be upregulated in the native myocardium following MI and associated with adverse remodeling of the ECM [26]. Our results show a two- to four-fold downregulation across all treatment groups, suggesting that some anti-fibrotic benefit may be observed upon stimulation with any Notch ligand, but Jag1 produces the most anti-fibrotic phenotype.

We also considered the secretion of pro-migratory factors that may attract other beneficial cell types, such as MSCs. The inflammatory chemokines CXCL10 and CXCL2 are both known to play a role in attracting mesenchymal stem cells, and both were strongly upregulated upon treatment with Jag1 as opposed to the other canonical ligands [27]. CXCL10 showed 12-fold greater expression while CXCL2 showed 8-fold greater expression in response to Jag1 stimulation, although these chemokines were also upregulated to a lesser degree in the other treatment groups. Based on this, we hypothesized that a transwell migration assay for bone marrow-derived hMSCs would demonstrate greater migration in response to the Jag1-treated conditioned media. However, although migration of MSCs was greater across all groups compared to our control, there were no significant differences between Jag1 and the other treatment groups. It is possible that other important cell types are more strongly attracted, and future studies will focus on determining if this is the case.

Finally, some of the most striking differences noted in our gene array relate to epigenetic regulators, such as the histone deacetylase (HDAC) and Mastermind-like (MAML) family of proteins. Deacetylation of histones promotes chromatin condensation, which in turn silences genes by making them inaccessible to the transcription complex [28,29] In this context, HDACs have been posited as negative regulatory elements in the Notch signaling pathway, reducing the overall level of Notch activation by chromatin condensation. HDACs 1, 3, 4, 7, 10, and 11 were all strongly downregulated in response to Jag1 stimulation, while the same HDACs were moderately upregulated in response to stimulation with Jag2, Dll1, and Dll4. This suggests a positive feedback loop upon stimulation with Jag1, whereby Notch activation is continuously promoted, whereas stimulation with the other ligands induces a negative feedback loop that makes the cells less responsive to Notch, likely leading to a more transient response. This is in keeping with the pattern of tip cell selection in endothelial cells, mediated by Notch, whereby Jag1 stimulates the formation of new tip cells, whereas stimulation with the other ligands inhibits tip cell formation, leading to a more quiescent stalk cell phenotype.

In summary, our findings suggest that while all four canonical Notch ligands are capable of activating Notch in vitro and producing some regenerative effects, stimulation with Jag1 remains the most viable option for hCPC-related therapies. Jag1 induces secretion of the important basement membrane proteins, Col4A1 and Col4A2, and produces the strongest VEGF signaling, particularly VEGFC. Additionally, stimulation with Jag1 produced the greatest chemotactic signaling, including chemokines CXCL2 and CXCL10, which are capable of recruiting cell types including endothelial cells and mesenchymal stem cells. Finally, stimulation with Jag1 downregulates the expression of HDAC family proteins, promoting a more open chromatin profile and, therefore, a more active phenotype compared to stimulation with the other three ligands.

## 4. Materials and Methods

**Figure 6:**
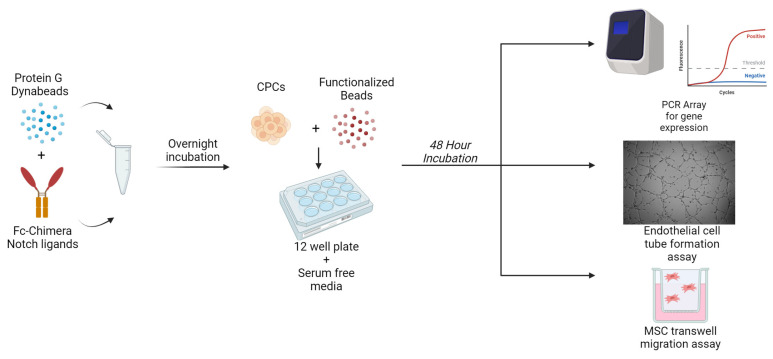
Illustrated design of the study. Protein G Dynabeads are functionalized overnight by incubation with Fc-conjugated Notch-activating ligands. Following functionalization, c-kit+ CPCs are incubated with functionalized beads in a well plate for 48 h. At the conclusion of this, RNA and conditioned media are collected to perform gene expression analysis via PCR array, endothelial cell tube formation assays, and a transwell assay to assess the migration of MSCs.

The design of this study is illustrated in Figure 6. Briefly, protein G Dynabeads (Thermo-Fisher Scientific, Waltham, MA, USA) were incubated overnight with Fc-conjugated Notch ligands. CPCs were then incubated with functionalized beads in a 12 well plate for a period of 48 h. At the conclusion of this period, conditioned media and RNA were harvested for the remaining experiments. 

### 4.1. CPC Isolation and Culture

Human c-kit+ cardiac progenitor cells were isolated from biopsy tissue graciously provided by our collaborators at Children’s Healthcare of Atlanta (CHOA). For this study, cells were isolated from donor tissue from four neonatal patients (age < 2 weeks) undergoing reparative surgery for hypoplastic left heart syndrome (HLHS). In the normal course of palliative surgery, the atrial appendage, a piece of vestigial tissue, was removed and donated with parental consent to our lab. C-kit+ cells were then isolated by enzymatic digestion with collagenase type-II, followed by a magnetic bead pulldown for c-kit using anti-CD117 microbeads (Miltenyi Biotech, Bergisch Gladbach, Germany). Following isolation, cells were maintained in Ham’s F12 media supplemented with 10% FBS, L-glutamine, and penicillin-streptomycin cocktail. Upon expansion, cells were frozen and cataloged as part of a library of patient-derived CPCs maintained by our laboratory.

### 4.2. Dynabead Preparation

Protein-G magnetic Dynabeads (Thermo-Fisher Scientific, Waltham, MA, USA) were functionalized by incubation with chimeric Notch-activating ligands, consisting of the full-length ligand fused to the Fc- region of human IgG, which binds with high affinity to protein G. Chimeric Fc-containing Jagged1, Jagged2, Dll1, and Dll4 ligands were obtained from R&D Systems/Biotechne (Minneapolis, MN, USA). As a control, normal human IgG was also obtained from R&D Systems/Biotechne. For each treatment group, 25 µL of Dynabeads was transferred to an Eppendorf tube and separated from the supernatant by a strong magnet. The supernatant was aspirated, and the beads were rinsed twice in sterile PBS + 0.1% Tween-20. For each group, 5 µg of the appropriate ligand was added to the Dynabeads and diluted in 100 µL of the PBS + 0.1% Tween solution. This concentration was chosen in order to completely saturate the beads, according to the theoretical binding capacity of Dynabeads as reported by the manufacturer. The beads were functionalized with constant rotation overnight at 4 °C (approximately 16 h total). Following the overnight functionalization, the beads were again separated from the supernatant by a strong magnet and rinsed twice in sterile PBS without tween. Finally, the beads were resuspended in 100 µL serum-free Ham’s F12 media before use in cell experiments.

### 4.3. Cell Treatment with Functionalized Dynabeads

C-kit+ CPCs were harvested at 70–80% confluence and resuspended in serum-free media at a concentration of 250,000 cells per 10 µL. Cell suspension was divided into 10 µL aliquots for each treatment group, to which 10 µL of the appropriate functionalized Dynabead solution was added. The combined Dynabeads and cell suspension were incubated under constant rotation in a cell incubator for 1 h. After this incubation, examination under a microscope showed nearly all the Dynabeads were able to bind preferentially to the cell membranes of the CPCs. The cells were then added to the wells of a pre-prepared 24-well plate containing 500 µL in each well of 10% FBS-supplemented Ham’s F12 media. After 24 h in culture, the media was exchanged for serum-free Ham’s F12 media in order to generate conditioned media free from serum and pro-angiogenic factors aside from those secreted by the cells themselves. After 48 h, conditioned media from each well was harvested and cells were lysed in TRIzol reagent (Thermo-Fisher Scientific, Waltham, MA, USA).

### 4.4. RNA Purification and RT-PCR

After the samples were lysed in TRIzol, RNA was purified from the samples using the standard manufacturer’s protocol. cDNA was generated from the resulting RNA by reverse transcription, which was then used to perform RT-PCR. Individual RT-PCR experiments on Notch downstream targets HEY1 and HES1 were performed using an Applied Biosciences StepOnePlus RT-PCR thermal cycler and using Applied Biosciences SYBR Green Mastermix and the following primers: HEY1 (fwd: CCGCTTCGTGTTCGCCTGGT, rev: TGCTGCCTGTGAGGGTGTCG), HES1 (fwd: CCGAGCGTGTTGGGGAAATAC, rev: GTTGATCTGGGTCATGCAGTTGG), and GAPDH (housekeeping gene, fwd: GTGGACCTGACCTGCCGTCT, rev: GGAGGAGTGGGTGTCGCTGT). Microarray data were generated using two separate TaqMan microarray plates, TaqMan Human Notch Signaling and Human Angiogenesis arrays from Applied Biosciences (Waltham, MA, USA). For the arrays, N = 4 cDNA samples corresponding to the four patients analyzed were pooled for each treatment group, approximately 20 ng of cDNA for each well. Arrays were prepared according to the manufacturer’s instructions using TaqMan Fast Universal PCR master mix (Applied Biosciences) as suggested.

### 4.5. Endothelial Cell Tube Formation Assay

Human cardiac endothelial cells (CECs) were first cultured in EBM-2 basal media supplemented with an EGM BulletKit (Lonza, Basel, Switzerland) in standard T-75 tissue culture flasks coated with 0.1% gelatin solution. Ibidi µ-slide angiogenesis chambered slides were prepared according to the manufacturer’s instructions by pipetting 10 µL thawed Matrigel into the inner well of each chamber and allowing it to cure for 30 min in an incubator at 37 °C. A total of 50 µL of conditioned media was placed in the outer well of each chamber. CECs at approximately 90% confluence were trypsinized, counted, and resuspended in serum-free media. A total of 10,000 CECs were added to each chamber and left in the incubator for 5 h. After 5 h, the conditioned media was aspirated, and the cells were stained for 20 min with Calcein-AM before visualization with an Olympus FV-1000 fluorescence microscope (Olympus Corporation, Tokyo, Japan). Representative images of each well were taken at 4× magnification. Subsequent analysis was performed using ImageJ Version 1.54J with the angiogenesis analyzer plugin.

### 4.6. hMSC Migration Assay

Human nasal polyp-derived MSCs (hMSCs) were first cultured in DMEM media supplemented with 10% FBS, L-glutamine, and penicillin-streptomycin cocktail. hMSC migration in response to secreted factors in the conditioned media was assessed using a Corning Transwell assay (Corning Life Sciences, Corning, NY, USA) and using 8.0 µM pore size transwell inserts in a 24-well plate format. A total of 300 µL of conditioned media was added to the bottom well of each assay well, while 200 µL of cell suspension, containing approximately 125,000 hMSCs, was added to the top well of each transwell insert. The plate was incubated for 6 h in a cell culture incubator, after which time media from the upper wells of the inserts was removed using cotton swabs, and the inserts were placed in a new well plate containing 300 µL TrypLE Express trypsin solution with EDTA in order to dissociate cells that had migrated through the membrane. After five minutes, the transwell inserts were removed, and an equal volume of serum-supplemented media was added in order to inactivate the trypsin. The contents of each well were transferred to Eppendorf tubes and centrifuged to pellet-migrated cells. Simultaneously, reserved cells that were not used in the transwell assay were loaded into identical Eppendorf tubes in quantities ranging from 0 to 125,000 in order to generate a standard curve for quantification. Cells were stained with CMRA orange dye, and fluorescence was measured using a Biotek Synergy II fluorescence plate reader (Agilent Technologies, Santa Clara, CA, USA) using a 530/590 nm excitation/emission filter cube.

## Data Availability

The raw data supporting the conclusions of this article will be made available by the authors on request.
